# Tai Chi Training may Reduce Dual Task Gait Variability, a Potential Mediator of Fall Risk, in Healthy Older Adults: Cross-Sectional and Randomized Trial Studies

**DOI:** 10.3389/fnhum.2015.00332

**Published:** 2015-06-09

**Authors:** Peter M. Wayne, Jeffrey M. Hausdorff, Matthew Lough, Brian J. Gow, Lewis Lipsitz, Vera Novak, Eric A. Macklin, Chung-Kang Peng, Brad Manor

**Affiliations:** ^1^Division of Preventive Medicine, Osher Center for Integrative Medicine, Brigham and Women’s Hospital, Boston, MA, USA; ^2^Harvard Medical School, Boston, MA, USA; ^3^Department of Neurology, Center for the Study of Movement, Cognition, and Mobility, Tel Aviv Sourasky Medical Center, Tel Aviv University, Tel Aviv, Israel; ^4^Institute for Aging Research, Hebrew Senior Life, Boston, MA, USA; ^5^Department of Neurology, Beth Israel Deaconess Medical Center, Boston, MA, USA; ^6^Biostatistics Center, Massachusetts General Hospital, Boston, MA, USA; ^7^Division of Interdisciplinary Medicine and Biotechnology, Beth Israel Deaconess Medical Center, Boston, MA, USA; ^8^Center for Dynamical Biomarkers and Translational Medicine, National Central University, Chungli, Taiwan

**Keywords:** Tai Chi, gait analysis, dual task performance, falls and fall risk prevention, cognition

## Abstract

**Background:**

Tai Chi (TC) exercise improves balance and reduces falls in older, health-impaired adults. TC’s impact on dual task (DT) gait parameters predictive of falls, especially in healthy active older adults, however, is unknown.

**Purpose:**

To compare differences in usual and DT gait between long-term TC-expert practitioners and age-/gender-matched TC-naïve adults, and to determine the effects of short-term TC training on gait in healthy, non-sedentary older adults.

**Methods:**

A cross-sectional study compared gait in healthy TC-naïve and TC-expert (24.5 ± 12 years experience) older adults. TC-naïve adults then completed a 6-month, two-arm, wait-list randomized clinical trial of TC training. Gait speed and stride time variability (Coefficient of Variation %) were assessed during 90 s trials of undisturbed and cognitive DT (serial subtractions) conditions.

**Results:**

During DT, gait speed decreased (*p* < 0.003) and stride time variability increased (*p* < 0.004) in all groups. Cross-sectional comparisons indicated that stride time variability was lower in the TC-expert vs. TC-naïve group, significantly so during DT (2.11 vs. 2.55%; *p* = 0.027); by contrast, gait speed during both undisturbed and DT conditions did not differ between groups. Longitudinal analyses of TC-naïve adults randomized to 6 months of TC training or usual care identified improvement in DT gait speed in both groups. A small improvement in DT stride time variability (effect size = 0.2) was estimated with TC training, but no significant differences between groups were observed. Potentially important improvements after TC training could not be excluded in this small study.

**Conclusion:**

In healthy active older adults, positive effects of short- and long-term TC were observed only under cognitively challenging DT conditions and only for stride time variability. DT stride time variability offers a potentially sensitive metric for monitoring TC’s impact on fall risk with healthy older adults.

## Introduction

The ability to walk while simultaneously performing a secondary cognitive task – commonly referred to as a dual task (DT) – is essential to many activities of daily living such as successful ambulation while navigating complex environs and conversing with others. Increasing evidence from clinical practice, epidemiological studies, and clinical trials show that postural control, gait health, and cognition are interrelated in older adults (Montero-Odasso et al., 2012). Observational studies have reported that the magnitude of the decrement in gait performance during a DT (i.e., the DT “cost”) is higher in elderly fallers as compared to non-fallers (Springer et al., 2006), and recent long-term prospective epidemiological studies demonstrated that gait performance, and especially stride-to-stride variability, during a DT may be a particularly sensitive predictor of falls in older adults (Herman et al., 2010; Mirelman et al., 2012). The importance of cognition in gait performance and postural control is further supported by a growing body of studies employing a variety of neuroimaging (e.g., fMRI, fNRIS) and neurostimulation techniques (e.g., tDCS, TMS), which suggest that gait and executive function may share a network of brain regions in the frontal and parietal cortex (Gatts and Woollacott, 2006, 2007; Halsband and Lange, 2006; Mirelman et al., 2014; Zhou et al., 2014), often referred to as the fronto-parietal executive control network (Tessitore et al., 2012; Markett et al., 2014).

Growing appreciation of the interdependence of cognitive and postural control processes has led to search for multimodal interventions combining motor and cognitive training for improving gait and preventing falls (Mirelman et al., 2013; Kayama et al., 2014; Shema et al., 2014). Tai Chi (TC) is a multi-component mind–body exercise that is growing in popularity, especially among older adults (Wayne and Fuerst, 2013). TC integrates training in balance, flexibility, and neuromuscular coordination with a number of cognitive components including – heightened body awareness, focused mental attention, imagery, multi-tasking, and goal-oriented training – which together may result in benefits to gait health and postural control, beyond conventional uni-modal exercise (Wayne et al., 2013). Evidence supports the idea that TC can improve balance and reduce fall risk in healthy and neurologically impaired older adults (McGibbon et al., 2004; Li et al., 2012; Manor et al., 2013), and may impact multiple aspects of gait health (McGibbon et al., 2005; Wu and Hitt, 2005; Wu and Millon, 2008; Vallabhajosula et al., 2014). Additionally, clinical and neurophysiological data indicate that TC may attenuate age-related cognitive decline, including executive function, which is critical to dynamic postural control (Wei et al., 2013; Hawkes et al., 2014; Wayne et al., 2014b). However, the potential for TC to reduce cognitive–motor interference, and specifically to improve gait performance during a DT activity, has not received much attention (Amano et al., 2013; Manor et al., 2014).

The current study evaluates the impact of both long- and short-term TC training on gait speed and stride time variability during both undisturbed (single task) walking and walking with a cognitive DT challenge. Long-term training effects were assessed through observational comparisons of TC naïve healthy older adults and an age-matched sample of expert TC practitioners. Short-term effects of TC training were assessed by random assignment of the TC naïve healthy adults to either 6 months of TC plus usual care or usual care alone. Based on research to date, we predicted that (1) TC experts would exhibit greater walking speed and reduced variability, compared to controls, and that group differences would be greater under DT challenges; (2) TC-naïve older adults randomly assigned to 6 months of TC would subsequently exhibit greater walking speed and reduced stride time variability; and (3) improvements in walking speed and reduced stride time variability observed over 6 months would be greater in those randomized to TC compared to a usual care control, with between-group differences being greater under DT challenges.

## Methods

### Study design

We employed a hybrid study design that included a two-arm randomized clinical trial (RCT) along with an additional observational comparison group. The Institutional Review Board at Beth Israel Deaconess Medical Center approved this study. The RCT component of this study was registered at clinicaltrials.gov (NCT01340365). Gait outcomes reported herein are a subset of a larger battery of assessed outcomes including balance, cardiovascular, and cognitive outcomes. These latter outcomes are reported independently (Wayne et al., 2013, 2014a).

### Randomized trial design

Sixty healthy older adults, aged 50–79, were randomized 1:1 to receive 6 months of TC training in addition to usual health care, or to usual health care alone (control group). Study participants randomized to usual care were offered a 3-month course of TC as a courtesy following the trial. Randomization was stratified by age (50–59, 60–69, 70–79 years) and utilized a permuted-blocks randomization scheme with randomly varying block sizes. Randomization was performed by the study statistician. All outcomes were assessed at baseline and 6 months, i.e., after completing 6 months of training. The primary staff overseeing assessment and analyses of the gait-related outcomes was blinded to treatment assignment. Recruitment spanned from March 2011 to March 2013. All follow-up procedures were completed by September 2013. Analysis was performed in 2014. Further details related to the design of the RCT component of this study are reported elsewhere (Wayne et al., 2013).

### Recruitment for the randomized trial targeting community-dwelling healthy adults

Inclusion criteria were (1) age 50–79 years; (2) living within the Greater Boston area; and (3) willing to adhere to a 6-month TC training protocol. Exclusion criteria were (1) chronic medical condition including cardiovascular disease, stroke, active cancer; neurological conditions; or significant neuromuscular or musculoskeletal conditions requiring chronic use of pain medication; (2) acute medical condition requiring hospitalization within the past 6 months; (3) self-reported inability to walk continuously for 15 min unassisted; (4) regular TC practice within past 5 years; and (5) regular participation in physical exercise on average 4 or more times per week. Interested individuals underwent both an initial phone screen and an in-person screen at the BIDMC Clinical Research Center. Eligible individuals provided written informed consent and underwent baseline testing prior to randomization.

Participants within both groups were encouraged to follow usual health care as prescribed by their primary care physicians. Participants in the TC group received 6 months of TC training in addition to usual care. All TC interventions were administered pragmatically at one of five pre-screened TC schools within the Greater Boston area that met specific guidelines described elsewhere (Wayne et al., 2013). Study participants were asked to attend, on average, two classes per week over the 6-month intervention. They were also asked to practice a minimum of 30 min, two additional days per week. Attendance at TC classes was recorded by instructors and home practice was tracked by participants using a weekly practice log. Participants that reported attending a minimum of 70% of all classes and completing 70% or more of prescribed home practice between each study visit were considered compliant or “per-protocol.” Adverse events were systematically collected from participants and instructors and are reported elsewhere (Wayne et al., 2014a).

### Non-randomized comparison group: Tai Chi experts

Twenty-seven healthy older adults (age 50–79 years) currently engaged in an active TC training regimen, each with over 5 years of TC practice, were recruited for a single observational visit. No limitation was set on TC style. Eligibility and screening procedures for TC experts were identical to those for healthy adults enrolled in the RCT.

### Measurements

Participants reported to the Beth Israel Deaconess Medical Center’s Clinical Research Center (Boston, MA, USA) where they underwent in-person screening procedures. Participants with a mini mental state examination (MMSE) score ≥24 and no abnormal findings on an ECG were eligible to participate in the study. All outcome measurements were assessed at the Syncope and Falls in the Elderly (SAFE) laboratory at Beth Israel Deaconess Medical Center. Outcomes related to gait reported here were part of a larger battery of tests that lasted an average of 3.5 h. Changes in DT gait speed and gait variability from baseline to 6 months were *a priori*-defined outcome measures of primary interest.

#### Assessment of Gait

Gait was assessed along a 75-m long unobstructed hallway. Subjects were instructed to walk at their normal preferred walking pace and make wide turns at the ends of the hallway. Two 90 s walking trials were completed: undisturbed with no superimposed cognitive task (i.e., single task walking) and walking while completing a cognitive DT. The cognitive DT consisted of a serial subtraction exercise counting backwards by threes beginning at 500. Ultrathin, force-sensitive resistor footswitches were placed on each subject’s heel and toe to capture the temporal parameters of gait. Data were collected wirelessly at 1500 Hz with DTS Data Acquisition Software (Noraxon, Scottsdale, AZ, USA). The data for each trial was exported and analyzed in Matlab (Mathworks, Natick, MA, USA) to determine initial and final foot contact times for each stride. Stride times were calculated for each gait cycle as the time between initial heel strike of one foot and the subsequent heel strike of that same foot. Average gait speed was measured using the total distance walked during the trial. Stride time variability was calculated from the stride time time-series as the coefficient of variation (CV, 100 multiplied by the SD of the stride times divided by the mean of each subject’s stride times). To calculate these measures, the first five strides were removed to minimize potential gait initiation effects. A median filter was applied to the stride time time-series to remove large outliers, typically a result of turns at the ends of the hallway. This ensured that the steady state variability was analyzed for each time series. Average stride time and stride time variability was determined from the resultant time series.

Dual task costs were calculated using both absolute and proportional measures. Absolute DT costs (Abs. DT) were calculated for each participant as the difference in walking speed or stride time variability between undisturbed single task walking (ST) and DT walking. Proportional DT costs (% DT) were calculated for each participant’s walking speed or stride variability as: 100 × ((DT − ST)/ST). Both the number and the accuracy of serial subtractions during DTs were recorded.

#### Baseline Cognitive Function and Physical Activity

Global cognitive function at baseline was assessed with the MMSE. Executive cognitive function was assessed using the trail making test (TMT B and TMT B-A) (Bowie and Harvey, 2006; Sanchez-Cubillo et al., 2009). TMT B assesses the time required to connect a series of circles in an alternating sequence of numbers and letters (e.g., 1-A-2-B-3-C). TMT B is considered to evaluate executive control, and is correlated with other executive function measures (Arbuthnott and Frank, 2000). TMT B is a sensitive indicator of overall neurological impairment and has good reliability (Bowie and Harvey, 2006). TMT A assesses the time required to connect a series of numbers. The difference between TMTB and A more accurately assesses executive function since it corrects for processing speed. Physical activity level was assessed using the physical activity status scale (PASS). Subjects were asked to estimate their general physical activity during the previous week using an 11-point scale (i.e., 0–10). The scale quantifies physical activity duration by a combination of the minutes of exercise per week and the intensity of this exercise (i.e., heavy, modest, or none) (Heil et al., 1995; Jackson et al., 1995).

#### Statistical Analysis

Cross-sectional measures of gait outcomes in TC experts and TC naïves were compared in a linear model controlling for age, gender, BMI, and physical activity and assuming equal variance across groups. For measures where the assumption of equal variance may have been violated, the larger of the two groups, the TC naïves, had greater estimated variance, leading to potential overestimates of pooled variance and conservative estimates of effect sizes and *p*-values. Inference was unchanged when applying an equivalent generalized least squares model allowing for variance heterogeneity between groups. Trajectories of gait performance over the 6-month intervention were compared between naïve participants randomized to TC or usual care using a random-slopes model with shared baseline. All longitudinal analyses were conducted according to the intention-to-treat paradigm. The model included fixed effect of time, time × treatment, age, gender, BMI, physical activity, and interactions between time and age, gender, BMI, and physical activity. The model included random participant-specific intercepts and slopes with unstructured covariance. The shared baseline assumption, enforced by omitting a treatment main-effect term, properly reflects the true state of the population sampled prior to randomization and has the advantage of adjusting for any chance differences at baseline (Zeger and Liang, 1986). Treatment-group differences and adjusted means for a male participant with mean age, BMI, and physical activity were estimated as well as their 95% confidence intervals. Both cross-sectional and longitudinal effect size estimates were calculated using pooled baseline SDs after adjusting for age, gender, BMI, and physical activity (Feingold, 2013). All inferential tests were two-tailed with alpha set at 0.05. We chose to report comparison-wise *p*-values without adjustment for multiple comparisons to avoid inflating type II errors, recognizing that the nominal *p*-values underestimate the overall experiment-wise type I error rate. Our results are intended as hypothesis generating, not definitive tests of efficacy for TC on any specific measure of gait. No comparisons were significant after a step-down Bonferroni adjustment for multiple comparisons. All analyses were performed in SAS (version 9.3, SAS Institute, Cary, NC, USA).

#### Sample Size Considerations

For cross-sectional comparisons, we estimated that a sample size of 27 TC Expert and 60 TC naïve subjects would provide power to detect an effect size of 0.63. For the randomized trial, we estimated that the sample of 60 participants randomized 1:1, the study would have 80% power to detect a main effect of treatment if the true effect size was at least 0.74 based on a two-tailed test at *p* < 0.05.

## Results

### Baseline characteristics and study flow

Demographic characteristics of the TC experts (*n* = 27) were well-matched with the older TC naïve group with respect to average age and cognitive status as measured by MMSE and trial making B test. Compared with naïve older controls, experts included a slightly greater proportion of men and Asians, had lower BMI’s, and higher levels of physical activity (see Table 1). TC experts reported an average of 24.6 ± 12 years of TC training experience (median: 20 years, range 10–50 years). Approximately equal numbers reported Yang (*n* = 12) and Wu (*n* = 15) style TC as their primary training systems; however, all reported having training experience in others styles of TC-related internal and external martial arts (e.g., kung fu, bagua) and/or mind–body practices (e.g., yoga, meditation).

**Table 1:** **Baseline characteristics**.

	Randomized groups		Observational group
	Usual care (*n* = 29)	Tai Chi (*n* = 31)	Tai Chi experts (*n* = 27)
**Age**
AVG ± SD	64.45 ± 7.42	63.94 ± 8.02	62.78 ± 7.57
**Gender *n* (%)**
Male	11 (37.9%)	9 (29%)	13 (48.1%)
Female	18 (62.1%)	22 (71%)	14 (51.9%)
**Race *n* (%)**
White	26 (89.7%)	29 (93.5%)	22 (81.5%)
African-American	3 (10.3%)	0 (0%)	1 (3.7%)
Asian	0 (0%)	2 (6.5%)	4 (14.8%)
**Ethnicity *n* (%)**
Non-Hispanic/Non-Latino	29 (100%)	30 (96.8%)	26 (96.3%)
Hispanic/Latino	0 (0%)	1 (3.2%)	1 (3.7%)
**Education (years)**
AVG ± SD	16.19 ± 3.03	17.13 ± 3.41	18.44 ± 3.34
**Mini mental state exam (MMSE)**
AVG ± SD	29.21 ± 0.82	29.03 ± 1.17	29.07 ± 1.11
**Trail making B (s)**
AVG ± SD	59.93 ± 20.84	59.69 ± 22.03	53.07 ± 22.4
**Trail making B–A (s)**
AVG ± SD	29.54 ± 18.58	30.26 ± 20.01	28.09 ± 19.65
**Body mass index (BMI; kg/m^2^)**
AVG ± SD	26.54 ± 5.83	26.38 ± 5.19	23.54 ± 2.35^a^
**Physical activity level^b^**
AVG ± SD	4.0 ± 2.0	4.0 ± 2.0	6.0 ± 2.0^a^

*^a^Cross-sectional comparisons between Tai Chi experts and Tai Chi naïve adults at baseline differed significantly (*p* < 0.05) in BMI and physical activity level*.

*^b^4 = Run about 1 mile/week OR walk about 1.3 miles/week OR spend about 30 min/week in comparable physical activity; 6 = run about 6–10 miles/week OR walk 7–13 miles/week OR spend 1–3 h/week in comparable physical activity*.

Tai Chi naïve adults randomized to TC plus usual care or usual care alone were comparable at baseline (see Table 1). For all variables, values for the subset of participants that were found to be “per-protocol” were comparable to those in the larger sample thereby minimizing potential sources of bias in *post hoc* comparisons between control and TC compliant groups.

**Figure 1:**
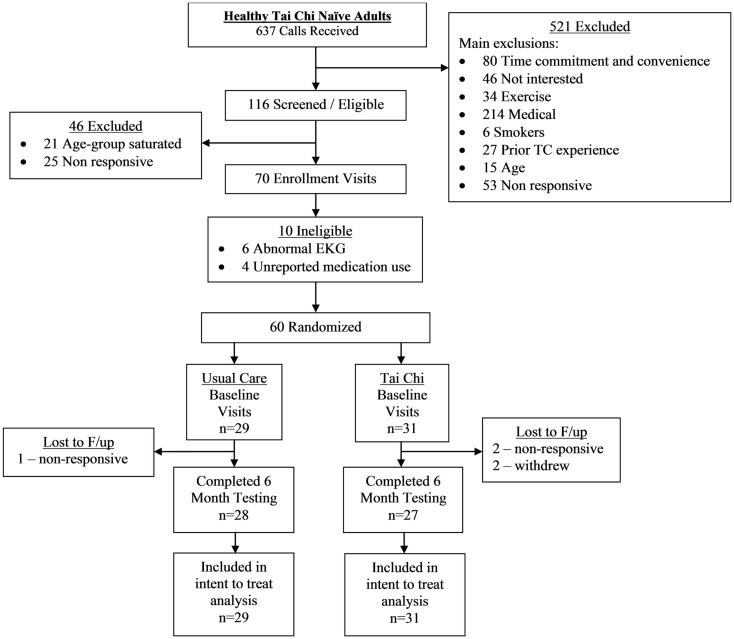
**Participant flow through the randomized trial sub-study**.

A CONSORT flowchart detailing study recruitment, randomization, and retention for the randomized trial component of the study is shown in Figure 1. Sixty healthy adults were successfully screened and enrolled, and 97% (28/29) and 87% (27/31) of individuals in the usual care and TC group completed the primary 6-month follow-up assessment, respectively.

Adherence to the TC protocol was variable. Two participants in the TC group formally withdrew participation due to time commitments and an unrelated injury. Of the remaining 29 participants in the TC group, 21 (72%) were per-protocol – defined as attending 70% of classes and completing 70% of required home practice over the entire course of the trial (mean and median TC exposure hours were 59.9 and 62.4 h, respectively).

For those randomized to TC, 13 subjects were trained at a TC school teaching Yang style and the remaining 18 subjects were trained at a school teaching Wu style.

### General effects of dual task challenges

Compared to undisturbed walking, gait speed decreased during DT walking in both TC expert (*p* = 0.003) and TC naïve (*p* < 0.001) adults. Stride time variability also increased with the addition of a DT in both TC expert (*p* = 0.004) and TC naïve (*p* < 0.001) adults (see Table 2).

**Table 2:** **Cross-sectional comparison of gait parameters for Tai Chi expert and Tai Chi naïve older adults during undisturbed single task (ST) and dual task (DT) walking**.

Outcome measure	Tai Chi expert (*n* = 27)	Tai Chi naïve (*n* = 60)	Between groups		
	Mean (95% CI)	Mean (95% CI)	Effect size	Mean difference (95% CI)	*p*-Value
Gait speed ST (m/s)	1.12 (1.1, 1.2)	1.12 (1.1, 1.2)	0.008	−0.001 (−0.08, 0.07)	0.97
Gait speed DT (m/s)	1.01 (1.0, 1.1)^a^	0.97 (0.9, 1.1)*	0.28	0.041 (−0.03, 0.1)	0.26
Stride time variability ST (CV %)	1.75 (1.5, 2.0)^b^	1.90 (1.8, 2.0)**	0.29	−0.15 (−0.4, 0.1)	0.25
Stride time variability DT (CV %)	2.11 (1.8, 2.4)	2.55 (2.3, 2.8)	0.57	−0.44 (−0.8, −0.5)	0.027
Abs. DT cost speed	−0.11 (−0.2, −0.1)	−0.15 (−0.2, −0.1)	0.34	0.042 (−0.02, 0.1)	0.18
% DT cost speed	−9.50 (−13.9, −5.1)	−13.24 (−16.1, −10.4)	0.34	3.74 (−1.7, 9.1)	0.17
Abs. DT cost variability	0.36 (0.03, 0.7)	0.65 (0.4, 0.9)	0.35	−0.29 (−0.7, 0.1)	0.16
% DT cost variability	23.49 (3.3, 43.7)	40.82 (27.7, 54.0)	0.35	−17.33 (−42.3, 7.6)	0.17

*Means and 95% confidence intervals predicted from linear model adjusting for age, gender, BMI, and physical activity*.

*ST, single task; DT, dual task; CV, coefficient of variation*.

^a^Within-group comparisons between undisturbed and DT walking speed among Tai Chi experts (*p* = 0.003) and *Tai Chi naïve (*p* < 0.001).

^b^Within-group comparisons between undisturbed and DT walking stride time variability among Tai Chi experts (*p* = 0.004) and **Tai Chi naïve (*p* < 0.001).

### Gait performance in Tai Chi experts vs. Tai Chi naïve older adults

**Figure 2:**
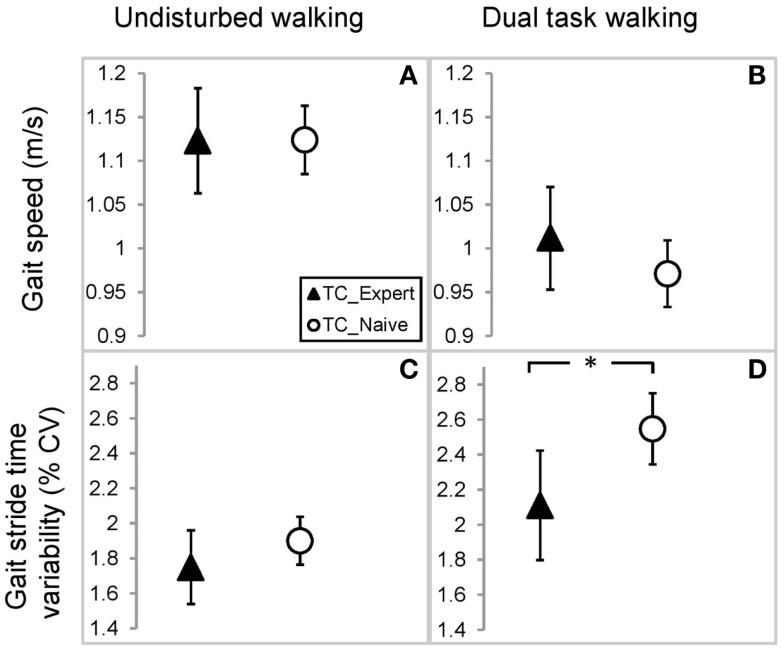
**Comparison of gait parameters between Tai Chi expert and Tai Chi naïve older adults**. Estimated mean and 95% confidence intervals for gait speed during undisturbed **(A)** and dual task walking **(B)**, and gait stride time variability during undisturbed **(C)** and dual task walking **(D)**. Values based on a linear mixed model accounting for variations in age, gender, BMI, and physical activity. *Indicates *p* < 0.05.

Linear models adjusting for variation due to age, gender, BMI, and activity revealed that average walking speeds were very similar in the TC naïve vs. TC expert groups during both undisturbed single task and DT walking. Group differences in these outcomes, as well as derived differences in absolute or percent DT costs, were not statistically significant (see Table 2; Figure 2). By contrast, stride time variability was lower in the TC expert vs. the TC naïve group, significantly so during DT walking (2.11 vs. 2.55%, respectively; *p* = 0.027). Absolute and percent DT costs of stride time variability also trended toward being lower in TC experts, but these differences were not statistically significant (recall Table 2). Of note, the two groups did not differ on performance of the serial subtractions during the DT walking trial. The number of serial subtractions attempted and their accuracy were 31 and 90%, respectively, in TC naïve group and 35 and 90%, respectively, in the TC expert group (*p* = 0.81). Age did not directly impact any gait variable, and treatment × age interactions for all cross-sectional comparisons were not statistically significant.

### The impact of short-term Tai Chi training on gait performance

Random-slopes model with shared baseline adjusting for variation due to age, gender, BMI, and activity also revealed trends toward reduced stride time variability following 6 months of TC. A small improvement in DT stride time variability (effect size = 0.2) was estimated with TC training, but no significant differences between groups were observed (see Table 3). Potentially important improvements after TC training could not be excluded in this small study. The estimated 95% CI for DT stride time variability includes a 20% improvement from short-term TC training. Effects of TC on stride time variability during quiet walking were negligible (ES < 0.1).

**Table 3:** **Longitudinal change in gait parameters for older adults randomly assigned to 6 months of Tai Chi vs. usual care during undisturbed single task (ST) and dual task (DT) walking**.

Outcome measure	Tai Chi (*n* = 31)			Usual care (*n* = 29)			Between groups		
	Baseline	6 months	Within-group *p*-value	Baseline	6 months	Within-group *p*-value	Effect size	Mean difference (95% CI)	*p*-Value
	Mean (95% CI)	Mean (95% CI)		Mean (95% CI)	Mean (95% CI)	
Gait speed ST (m/s)	1.12 (1.1, 1.2)	1.16 (1.1, 1.2)	0.074	1.12 (1.1, 1.2)	1.17 (1.1, 1.2)	**0.032**	0.076	−0.011 (−0.07, 0.05)	0.71
Gait speed DT (m/s)	0.97 (0.9, 1.0)	1.03 (1.0, 1.1)	**0.018**	0.97 (0.9, 1.0)	1.03 (1.0, 1.1)	**0.028**	0.016	0.002 (−0.06, 0.06)	0.94
Stride time variability ST (CV %)	1.96 (1.8, 2.1)	1.74 (1.5, 2.0)	0.11	1.96 (1.8, 2.1)	1.79 (1.5, 2.0)	0.22	0.079	−0.044 (−0.3, 0.3)	0.77
Stride time variability DT (CV %)	2.58 (2.3, 2.9)	2.29 (2.0, 2.6)	**0.035**	2.58 (2.3, 2.9)	2.46 (2.2, 2.8)	0.39	0.19	−0.17 (−0.5, 0.1)	0.27
Abs. DT cost speed	−0.15 (−0.2, −0.1)	−0.14 (−0.2, −0.1)	0.63	−0.15 (−0.2, −0.1)	−0.14 (−0.2, −0.1)	0.65	0.002	0.0 (−0.06, 0.06)	0.99
% DT cost speed	−12.92 (−16.7, −9.2)	−11.32 (−15.0, −7.7)	0.43	−12.92 (−16.7, −9.2)	−11.33 (−16.0, −6.7)	0.52	0.001	0.014 (−5.4, 5.4)	>0.99
Abs. DT cost variability	0.62 (0.3, 0.9)	0.61 (0.3, 0.9)	0.95	0.62 (0.3, 0.9)	0.62 (0.3, 0.9)	0.98	0.007	−0.007 (−0.4, 0.4)	0.97
% DT cost variability	39.48 (20.9, 58.1)	38.00 (20.7, 55.4)	0.90	39.48 (20.9, 58.1)	40.13 (22.1, 58.2)	0.96	0.037	−2.13 (−23.8, 19.6)	0.85

*Means and 95% confidence intervals predicted from shared baseline linear model adjusting for age, gender, BMI, and activity. No between-group comparisons (i.e., no tests of group × time interactions) were statistically significant. Within-group significant changes (*p* < 0.05) are highlighted in bold*.

*ST, single task; DT, dual task; CV, coefficient of variation*.

Both the TC and usual care group increased DT walking speed (*p* = 0.018 and *p* = 0.028), but the difference between groups in changes over 6 months were not significant (*p* = 0.94). No within- or between-group differences were observed for single task walking speed (see Table 3). *Post hoc* per-protocol analyses (i.e., limited to participants that were TC compliant) did not substantially change within- and between-group observed patterns and trends. Changes over 6 months in absolute and percent DT costs for both gait speed and stride time variability were small and largely uninfluenced by treatment assignment (recall Table 3). As in the cross-sectional comparisons, these groups did not differ on performance of the serial subtractions. The number of serial subtractions attempted and their accuracy at the final 6-month visit was 33 (95%) in usual care group and 33 (92%) in the Tai expert group (*p* = 0.58). Age did not directly impact longitudinal changes in any gait variable, and treatment × age interactions for all outcomes were not statistically significant.

## Discussion

To our knowledge, this study presents the first evidence that TC has the potential to positively impact DT stride time variability in healthy older adults. Cross-sectional comparisons revealed a significant degree of lower stride variability during dual tasking in TC experts, compared with TC naïve healthy older adults. Moreover, TC-naïve adults that were exposed to 6 months of TC training exhibited within-group significant improvements in DT stride time variability. By contrast, we observed limited impact of TC on stride variability during quiet walking or of TC on gait speed during single task walking.

As DT stride variability is associated with falls in the elderly (Visser, 1983; Nakamura et al., 1996; Hausdorff et al., 1997, 2001; Mbourou et al., 2003; Springer et al., 2006; Herman et al., 2010; Mirelman et al., 2012), the observed effect of TC on DT stride variability in this study may help to explain the positive effects of TC on fall risk reported in other studies (Logghe et al., 2010; Gillespie et al., 2012). Our results also suggest that DT stride variability may be a good discriminating metric for understanding the potential of TC to impact function in healthy and already active older adults. Finally, our findings support the value of research already underway to better understand the neurophysiological processes underlying how mind–body practices like TC impact cognitive–motor interactions (Li et al., 2014; Zheng et al., 2015).

Results of a recent 5-year prospective cohort study of healthy community-dwelling adults, ages 70–90 years and without gait impairments, provide context for the relevance of our findings. In adjusted models that accounted for age, gender, and fall history, baseline DT stride time variability (average CV was 2.97 ± 1.47%) was the only performance-based measure that predicted falls (Rate Ratio 1:11) (Mirelman et al., 2012). Although the DT stride variability in our relatively younger and healthier study population is moderately lower, we still found TC related improvements, with stride time variability decreasing by 11% following 6 months of TC training (from 2.58 to 2.29), and variability being 18% lower in TC experts vs. TC naives (2.11 vs. 2.58). These differences are clinically relevant and within a range that impacts future fall risk (Mirelman et al., 2012).

Prior studies evaluating the impact of TC on gait performance in older adults are quite variable in design and findings. We are not aware of any studies that evaluated the impact of TC on stride time variability and only a small number of studies have evaluated outcomes utilizing a DT paradigm. In one recent study of older adults (mean age 87.7 years) living in assisted living facilities, Manor et al. (2014) also reported an increase DT walking speed following 12 weeks of TC. However, unlike in our study, they also reported TC related increases in quiet walking speed. Our lack of an observed increase in quiet walking speed following TC parallels some other findings (Wolf et al., 2006; Zhang et al., 2006; Amano et al., 2013), although multiple studies have also reported TC-related increases in quiet walking speed (Yeh et al., 2004; Li et al., 2005; Shen et al., 2008; Li and Manor, 2010). Differences among these studies may be related to the age and health characteristics of the populations studied, and the different neuromuscular processes emphasized in different gait outcomes.

Our observed differences in outcomes based on stride time variability but not gait speed are not surprising. These features of gait have been shown to be independent and reflect different neuromotor processes (Hausdorff, 2005). Compared to gait speed, stride time variability reflects the control of the rhythmic stepping mechanism (Gabell and Nayak, 1984; Lord et al., 2013a), and has been characterized as an index of gait steadiness and control (Hausdorff et al., 1997, 2001; Verghese et al., 2009; Lord et al., 2013b). Inconsistency in gait rhythm, i.e., higher stride-to-stride variability, is a characteristic feature of gait in both Parkinson’s disease (Hausdorff et al., 2003; Lord et al., 2011) and multiple sclerosis (Socie and Sosnoff, 2013). Studies demonstrating improved TC motor control during gait in adults support our findings of reduced variability, and thus dynamic postural control, following long- and short-term TC training. For example, a randomized trial of frail elders reported that TC training improved neuromuscular coordination and the mechanism by which forward momentum is generated during gait initiation (Hass et al., 2004). Another trial in patients with vestibular disorders showed that TC was associated with reorganized lower extremity neuromuscular patterns, which appear to promote a faster gait and reduce hip compensatory movements (McGibbon et al., 2005).

Our finding that the impact of TC on gait was more apparent during DT vs. undisturbed walking conditions was also consistent with our hypotheses. DT-related changes in gait may result from interference caused by a competition between the attention demanded by gait and the attention demanded by a concomitant task, in our case, serial subtractions recited aloud (Verhaeghen et al., 2002; Woollacott and Shumway-Cook, 2002). Therefore, DT interference reflects a condition of more limited attentional resources, and thus is a more provocative condition for evaluating the impact of an intervention than is quiet walking. Even healthy young adults’ walk change their gait pattern under DT conditions (Srygley et al., 2009). Thus, we predicted that in the already active and healthy population of adults we studied, the impact of TC training would be most apparent in DT stride time variability, an outcome that challenges both the higher order process of gait rhythmicity and attentional competition. Our preliminary observations of both significant between-group differences in the comparison of TC experts vs. naives and within-group responses to 6 months of TC training, lends support to the hypothesis that TC enhances the attentional demands of walking. These findings also suggest that DT stride variability may be a sensitive and discriminating metric for evaluating the impact of interventions on cognitive–motor function in relatively healthy older adults. Other studies, including evaluations of cognitive–motor interactions in apparently asymptomatic prodromal Parkinson’s patients, have also reported DT stride time variability to be a discriminating metric (Mirelman et al., 2011).

The potential of TC to reduce cognitive–motor competition is supported by other studies in which more challenging DT activities have better discriminated potential cognitive–motor benefits of TC. For example, in a randomized trial comparing short-term TC training to more traditional balance training, when exposed to simulated slips (experimentally shifted supporting force plates) older adults exposed to TC showed reduced tibialis anterior reaction time and reduced occurrence of co-contraction with antagonist muscles (Gatts and Woollacott, 2006, 2007). In another cross-sectional comparison of older adults TC practitioners and healthy controls, participants were asked to step down from a 19-cm high platform and maintain a single leg stance with and without a concurrent cognitive task (Lu et al., 2013). While the TC group maintained better postural control under all conditions, improved performance was magnified under the DT conditions. Other studies have also reported TC-related benefits for stepping tasks that included mental distractions (Wu, 2012), and walking performance on obstacle courses that require motor planning (Zhang et al., 2011). However, there are studies that have reported no benefit of TC for tasks which require executive function (Hall et al., 2009).

### Limitations

Our study has a number of important limitations. First, samples for both the cross-sectional comparison and RCT were small, and could have resulted in type II errors. Because we considered this an exploratory study, we included statistical evaluations of outcomes without adjusting for multiple comparisons. When multiple comparisons were accounted for with Bonferonni adjustments, none of the outcomes were statistically significant. Findings in this study were intended to generate hypotheses to explore in future studies. Thus, the long- and short-term effects of TC on DT stride parameters in active healthy adults will need to be confirmed in larger, adequately powered studies. For our RCT, it is also possible that lack of more robust findings are due to the fact that 6 months of TC is insufficient training time to impact DT stride variability, and/or that more provocative DT challenges (e.g., more complex mental tasks and/or stride variability while negotiating turns or obstacles) are needed to observe any therapeutic impact of TC. Finally, our use of a non-active wait-list comparison group does not control for participant expectancy or psychosocial support afforded by participation in active TC programs. Future studies will require active comparison groups (e.g., alternative group exercise programs) that control for these factors.

With respect to our cross-sectional study, comparisons between TC experts and naïves may be confounded by differences between groups other than TC exposure. While linear models that included potential confounders (i.e., age, gender, BMI, physical activity) suggested an association with TC even after these factors were taken into account, other factors, including training in other martial arts could not be fully accounted for.

Another limitation of this study is lack of objective independent measures of proficiency in TC, which may have varied considerably especially in our expert TC group. In our cross-sectional comparison, TC experience among experts ranged from 10 to 50 years. A regression analysis indicated a slightly inverse relationship between years of training and DT stride variability, but this relationship was not statistically significant (*R*^2^ = 0.02 and *p* = 0.47). Similarly, per-protocol analysis of DT stride variability revealed marginally greater effect sizes than intent-to-treat analyses, but this effect was still not statistically significant. Future studies might benefit from using independent measures of TC skill or proficiency [e.g., Rosengren et al. (2003)], which would help guide analyses of direct associations between TC-related skills and therapeutic benefits. Finally, while our use of a pragmatic approach that included the evaluation of multiple TC styles affords a high level of generalizability, variation between styles may add additional heterogeneity in outcomes, resulting in further reduced power for a given sample size (Macpherson, 2004).

## Conclusion

In healthy active older adults, trends toward positive TC effects on gait were most obvious only under cognitively challenging DT conditions, and only for stride time variability. DT stride variability offers a potentially sensitive metric for monitoring the impact of TC on fall risk with healthy aging. These findings also support the value of neurophysiological research evaluating how mind–body exercises like TC impact cognitive–motor interactions and confirm previous findings, which suggest that TC reduces the risk of falls in older adults (Wolf et al., 1996; Gillespie et al., 2012). Future adequately powered studies are required to confirm these preliminary findings.

## Author Contributions

PW, JH, LL, CP, and BM conceived and designed the study. ML acquired the study data with oversight from VN. ML and BG analyzed the study data. BG and EM performed statistical analysis. PW drafted and revised the manuscript. All authors reviewed the manuscript and approved the final version.

## Conflict of Interest Statement

Peter M. Wayne is the founder and sole owner of the Tree of Life Tai Chi Center. Peter M. Wayne’s interests were reviewed and are managed by the Brigham and Women’s Hospital and Partners HealthCare in accordance with their conflict of interest policies. The Tree of Life Tai Chi Center did not provide payment or services for any aspect of this study. The other co-authors declare that the research was conducted in the absence of any commercial or financial relationships that could be construed as a potential conflict of interest.
